# Supplementary figures and images for: Activation of Protein Kinase A (PKA) signaling mitigates congenital hyperinsulinism associated hypoglycemia in the Sur1-/- mouse model
Source: PLoS One. 2020 Jul 31;15(7):e0236892. doi: 10.1371/journal.pone.0236892 (PMC7394442; doi:10.1371/journal.pone.0236892)

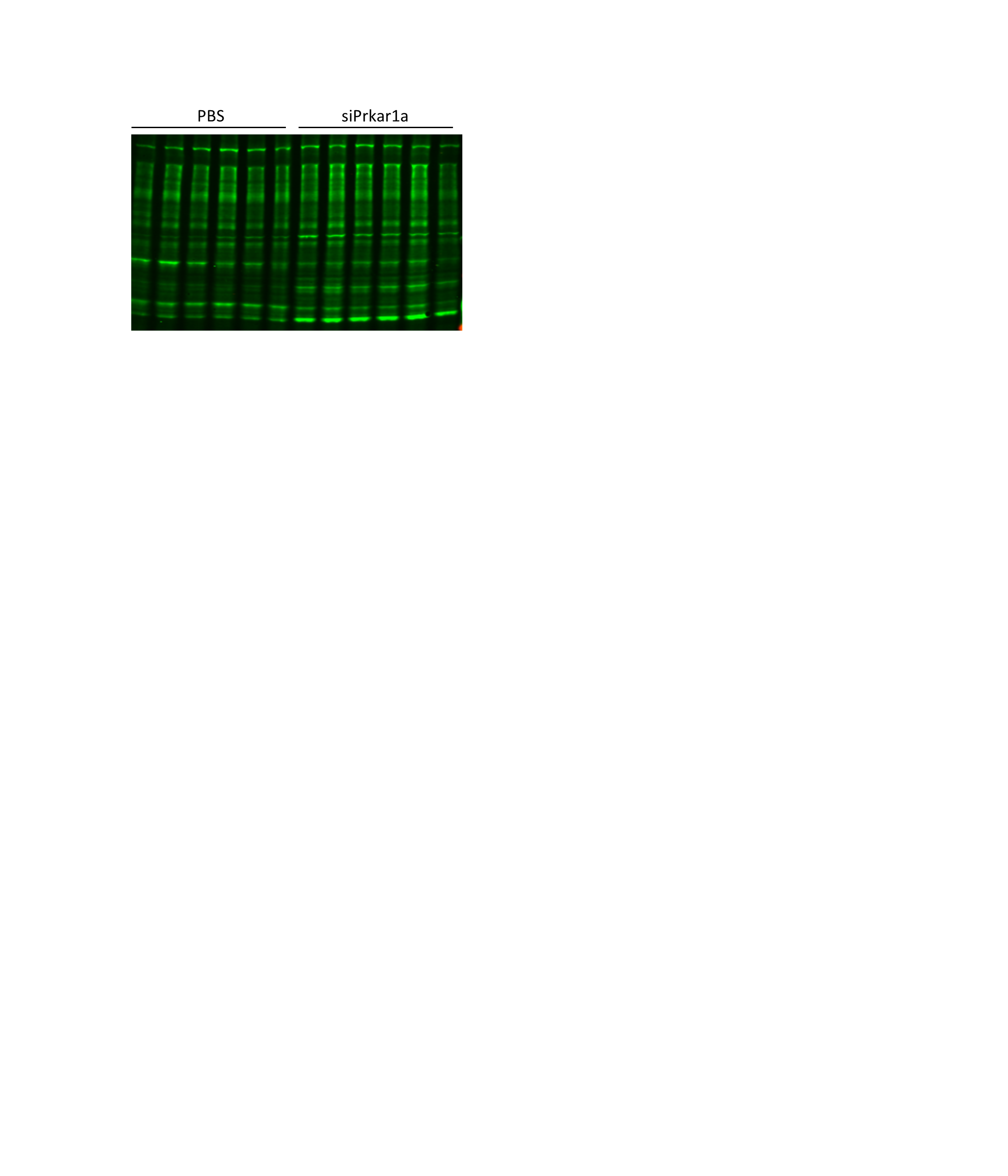

Supplement: S1 Fig — WT mice were injected with siRNA (AD-76410, 1mg/kg) directed against Prkar1a or PBS control every 2 weeks until liver tissue was collected 28 days post-injection of initial dose. Western blot analysis of phospho-PKA substrates was completed on the liver extracts (n = 6). (TIF) [file pone.0236892.s001.tif]

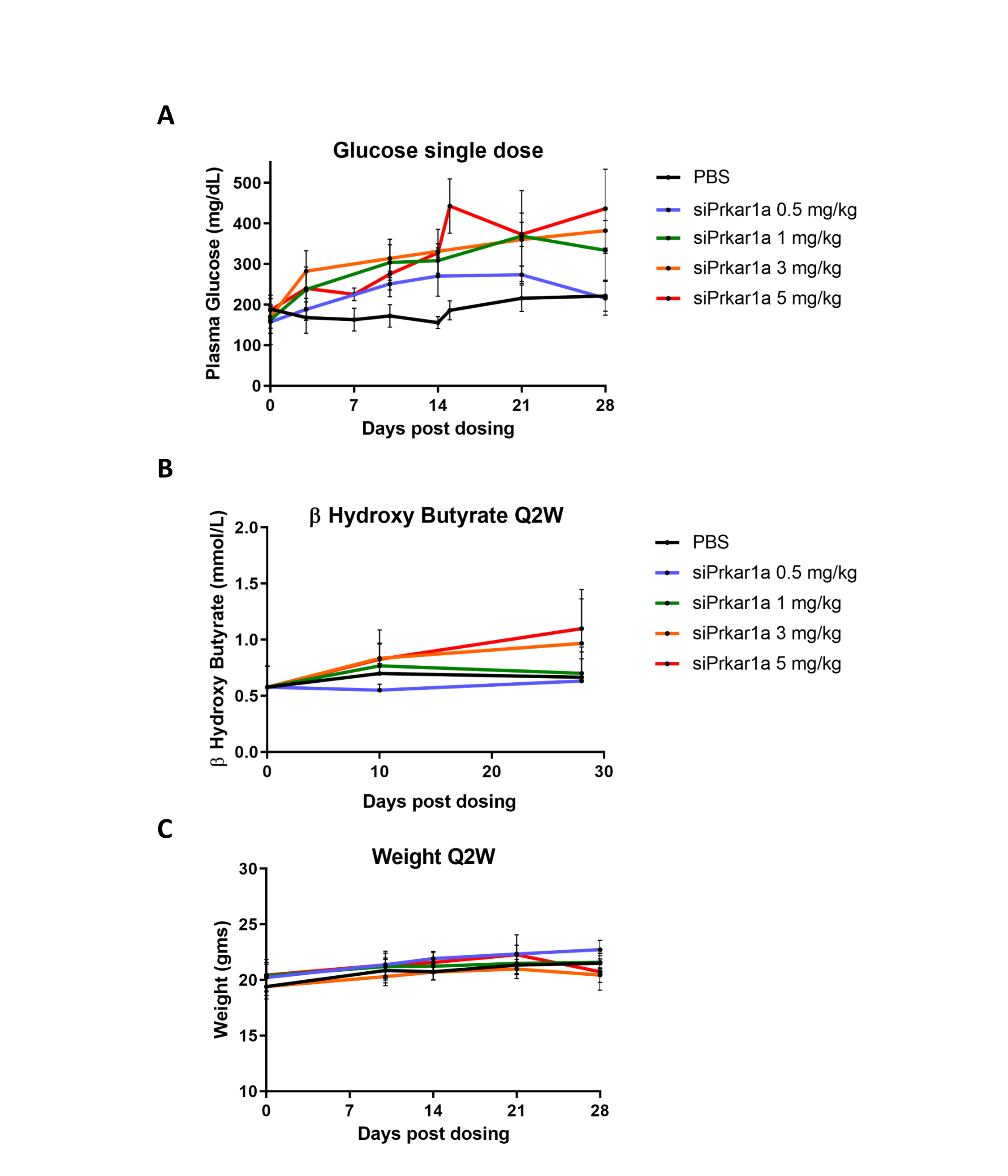

Supplement: S2 Fig — (A) WT mice were injected with siRNAs directed against Prkar1a once at day 0 at the denoted mg/kg dose and plasma glucose levels were assessed at 0, 3, 7, 10, 14, 21, and 28 days post dosing. (B) Mice were injected with siRNAs directed against Prkar1a every 2 weeks at the denoted mg/kg dose and plasma β hydroxyl butyrate levels were assessed at 0, 10, and 28 days post dosing. (C) Mice injected with siPrkar1a every 2 weeks (Q2W) were weighed at 0, 10, 14, 21, and 28 days post dosing. (n = 6 mice/group) Data represent mean +/- SEM. (TIF) [file pone.0236892.s002.tif]

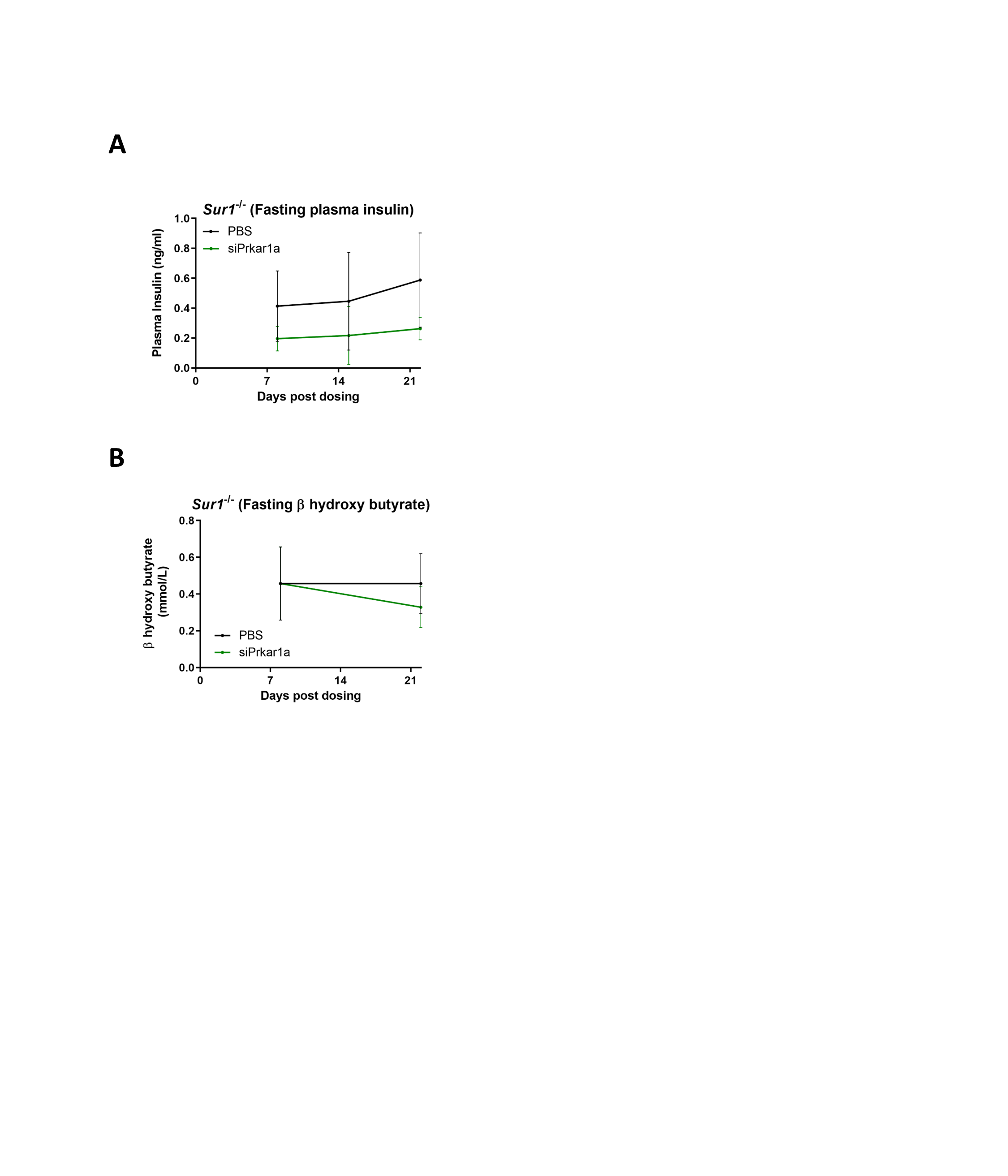

Supplement: S3 Fig — (A) In Sur1-/- mice, fasting plasma insulin levels were measured after a 16 hour overnight fast at 8, 15, and 22 days post dosing. (B) Fasting plasma β hydroxyl butyrate levels were measured after a 16 hour overnight fast at 8 and 22 days post dosing. (n = 7) Data represent mean +/- SEM. (TIF) [file pone.0236892.s003.tif]

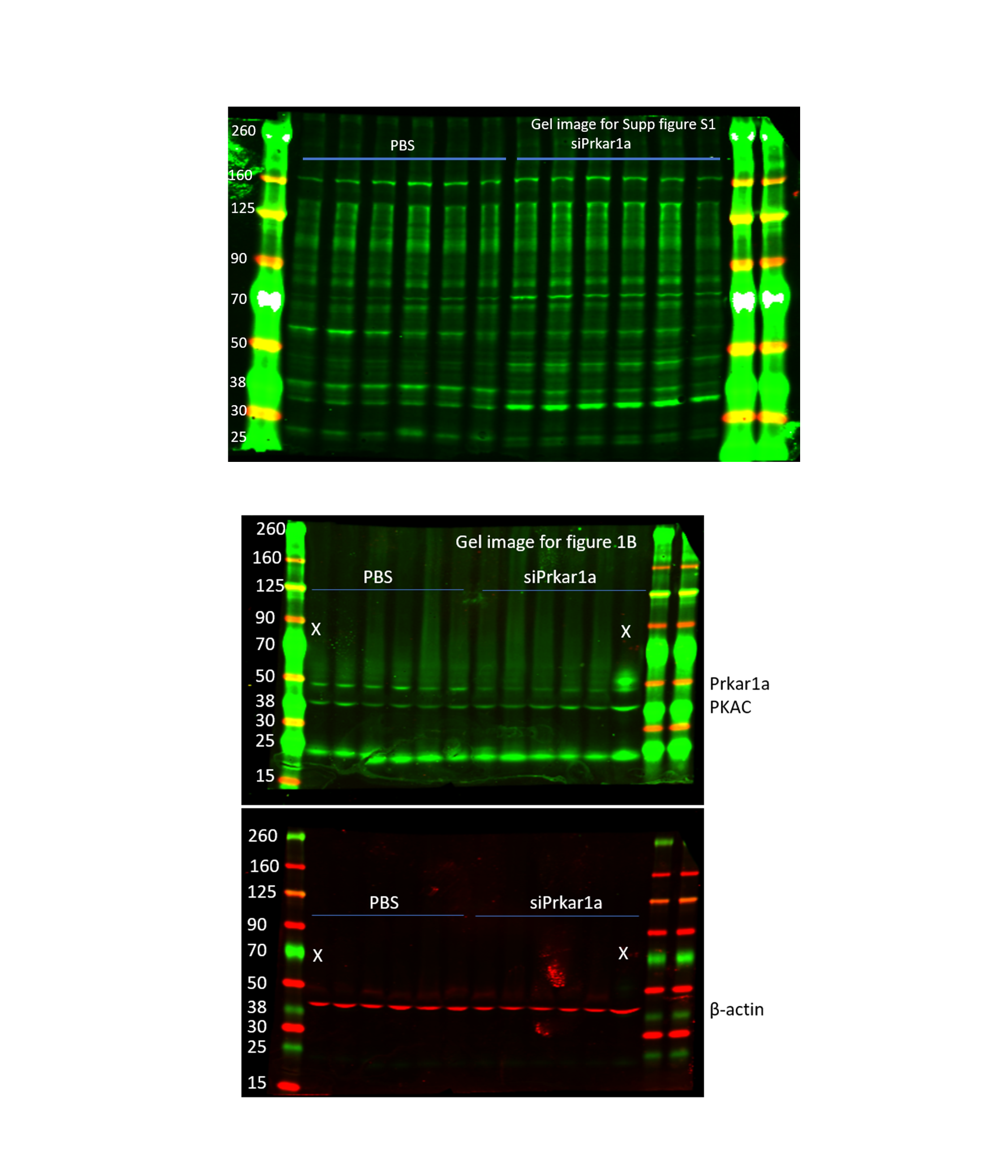

Supplement: S1 Raw images — (TIF) [file pone.0236892.s004.tif]
